# Actin Cytoskeleton Regulates Hippo Signaling

**DOI:** 10.1371/journal.pone.0073763

**Published:** 2013-09-11

**Authors:** Pradeep Reddy, Masashi Deguchi, Yuan Cheng, Aaron J. W. Hsueh

**Affiliations:** 1 Gene Expression Laboratory, Salk Institute for Biological Studies, La Jolla, California, United States of America; 2 Program of Reproductive and Stem Cell Biology, Department of Obstetrics/Gynecology, Stanford University School of Medicine, Stanford, California, United States of America; Institute of Molecular and Cell Biology, Biopolis, United States of America

## Abstract

Hippo pathway controls the organ size by modulating cell proliferation and apoptosis. However, the upstream regulation of hippo signaling by actin cytoskeleton is not clear. To elucidate the role of actin as an upstream regulator of Hippo signaling, the levels of F (filamentous)-actin in cells were elevated using jasplakinolide, an actin-stabilizing drug. Induction of F-actin formation in HeLa cells resulted in decreased phosphorylation of YAP, a key effector molecule for Hippo signaling. The activated YAP is localized to the cell nucleus and YAP increase was associated with increased expression of downstream CCN growth factors CCN1/CYR61 and CCN2/CTGF. The effect of the actin-stabilizing drug was blocked when YAP levels were suppressed in YAP “knock-down” cells. In summary, using an actin-stabilizing drug we show that actin cytoskeleton is one of the upstream regulators of Hippo signaling capable of activating YAP and increasing its downstream CCN growth factors.

## Introduction

Tissue growth and organ size are regulated by cell proliferation and cell death controlled by several developmental pathways [1]. Among them, the Hippo pathway was discovered in *Drosophila* using genetic screening [2], [3] and found to be a conserved tumor suppressor pathway in both *Drosophila* and vertebrates [4]–[10]. Mutations of various Hippo signaling genes were found in human cancers whereas tissue-specific deletion of different Hippo pathway genes in transgenic mice led to excessive tissue growth. Liver-specific deletion of Mst1 and 2 [8]–[10] or Sav1 increased liver size [9], [11] whereas cardiac-specific deletion of Sav1 led to enlarged heart [12].The key effector of Hippo pathway is YAP, a transcriptional coactivator whose phosphorylation by LATS kinases effects nuclear localization and increased activity [13]. Though the core components of Hippo signaling pathway are established, the upstream regulators are less clear.

Recent studies in *Drosophila* have shown the regulation of Hippo signaling by the actin cytoskeleton. Deletion of actin binding capping proteins or overexpressing constitutively active version of actin nucleation factor Diaphanous [14], [15] led to increased actin polymerization from globular (G)-actin to filamentous (F) form, leading to the disruption of Hippo signaling. In mammalian cells, changes in actin cytoskeleton due to mechanical cues were also shown to regulate YAP activity [16]–[18]. Moreover, LPA (lysophosphatidic acid) and S1P (sphingosine-1-phosphate), ligands for G-protein-coupled receptors, have been shown to regulate Hippo signaling mediated by changes in F-actin levels [19], [20]. Further, the activity of YAP is believed to be regulated by a particular F-actin structure, such as stress fibres or yet to be defined contractile actin network [21]. However, changes in actin polymerization were not shown in the mammalian cells studied and F-actin formation was mostly deduced from phalloidin staining analyses.

In the present study, we extended earlier studies by directly measuring G-actin and F-actin ratios following treatment of HeLa cells with an actin-stabilizing drug. We further analyzed the role of YAP in the induction of downstream CCN growth factors CTGF and CYR61.

## Results

### Jasplakinolide Induces Actin Polymerization

**Figure 1:**
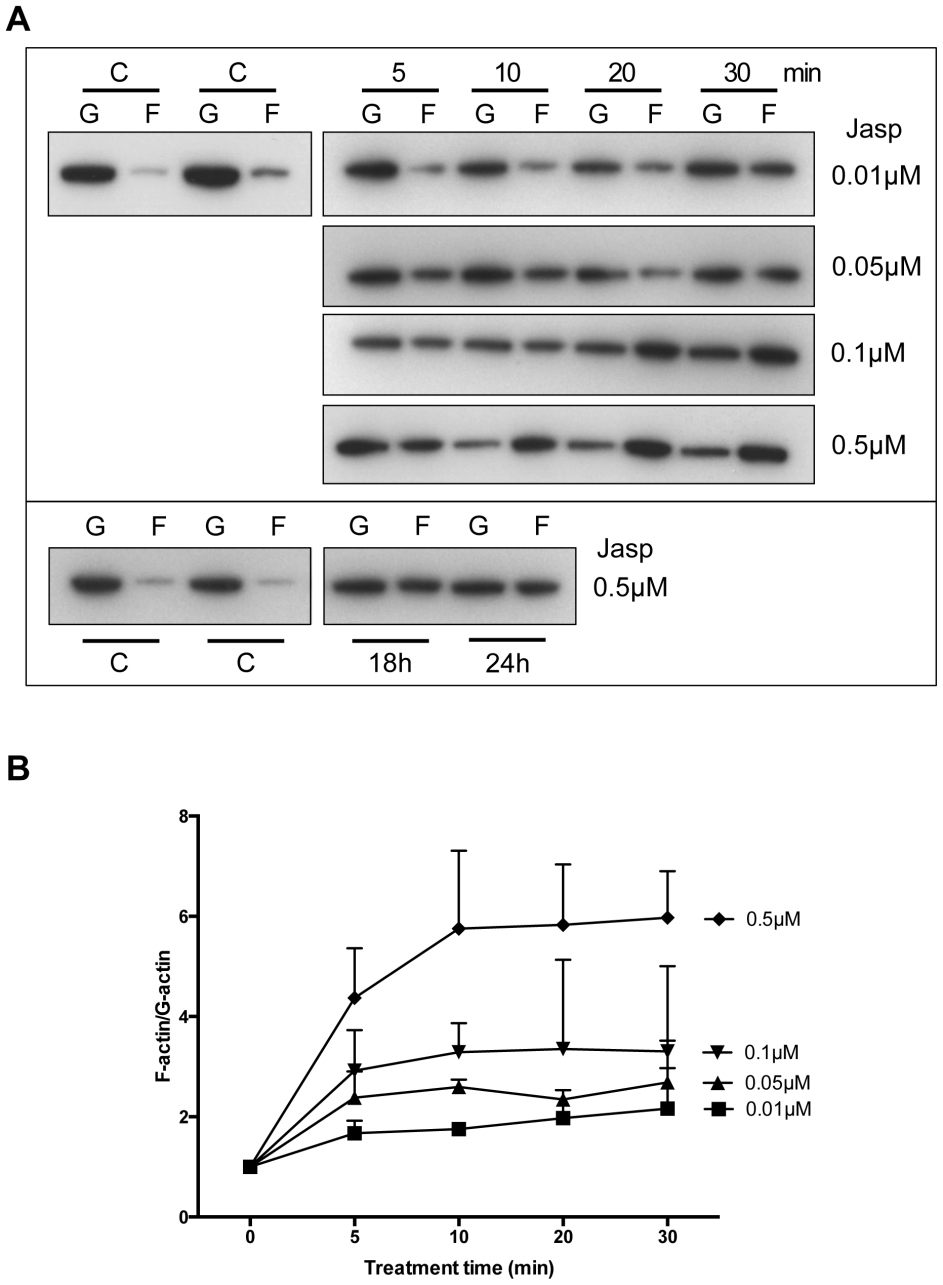
Jasplakinolide induces actin polymerization. A. HeLa cells were treated with different doses of jasplakinolide (Jasp). G-actin (G) and F-actin (F) fractions were separated as described in Materials and Methods. Equal amounts of G-actin and F-actin fractions were loaded onto SDS-PAGE gels and immunoblotting was done using the actin antibody. Representative data from three independent experiments is shown. B. Changes in the ratio of G-actin and F-actin from immune-blots were quantified using ImageJ. Data are from three independent experiments.

Jasplakinolide (Jasp), a naturally occurring cyclic peptide extracted from the marine sponge, *Jaspis johnstoni*, is a potent inducer of actin polymerization [22]. In order to quantify the conversion of monomeric G-actin to filamentous F-actin, the fractions of G-actin and F-actin after Jasp treatment were performed using HeLa cells. The conversion of G-actin to F-actin was time- and dose-dependent (Fig. 1A, upper panel). Increased conversion of G-actin to F-actin started at 5 min. after Jasp treatment and the F-actin levels reached a maximum level by 10 min. for 0.5 µM, whereas lower doses needed longer time (Fig. 1B). Moreover, the rate of conversion of F-actin back to G-actin was found to be very slow and F-actin levels were found to retain in the Jasp-treated cells even after 24 h of incubation (Fig. 1A, lower panel).

### Hippo Signaling is Disrupted Following Actin Polymerization

**Figure 2:**
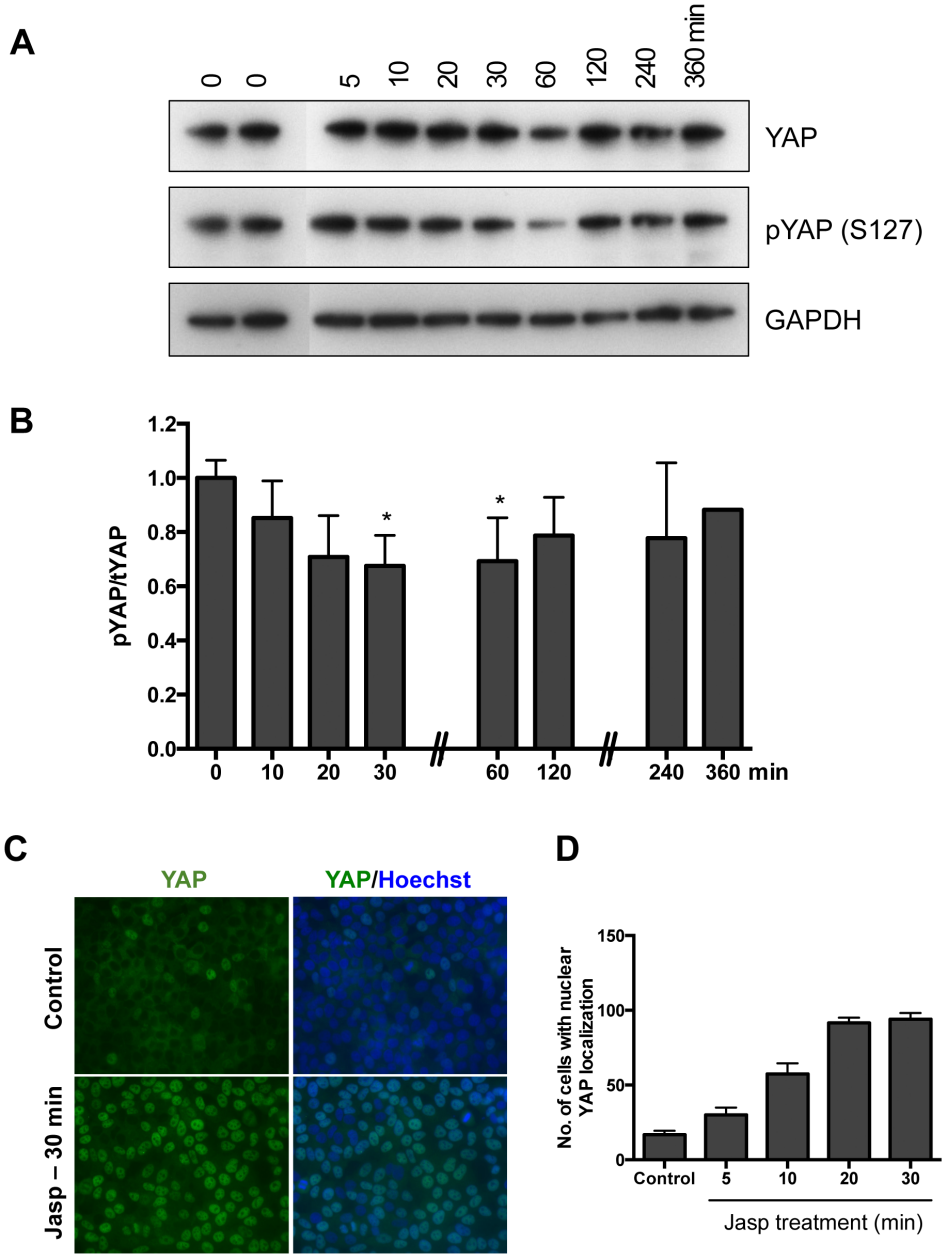
Actin polymerization lead to YAP de-phosphorylation and nuclear localization. A. The levels of pYAP are decreased after treatment with jasplakinolide. HeLa cells were treated with 0.5 µM jasplakinolide as described in Materials and Methods. Representative data from three to five independent experiments is shown. B. Changes in the ratio of pYAP/tYAP after jasplakinolide treatment. Densitometric analysis of immunoblotting analyses was done using ImageJ. Error bars represents mean ± SEM, n = 3. *P<0.05. C. Nuclear localization of YAP after Jasplakinolide treatment. HeLa cells were treated with 0.5 µM jasplakinolide and the cells were stained with the YAP antibody as described in Materials and Methods. D. Quantification of number of cells with nuclear YAP signals after jasplakinolide treatment at indicated time points.

YAP is a potent oncogene and one of the main effectors of the Hippo signaling pathway; YAP is activated in different human cancers [23], [24]. Hippo signaling controls the activity of YAP by phosphorylation to retain it in the cytoplasm. Decreases in YAP phosphorylation led to its nuclear localization, thus increasing YAP’s activity as a co-transcriptional factor [25]. To determine the effects of actin polymerization on Hippo signaling, changes in YAP phosphorylation were analyzed. Treatment with Jasp reduced the phosphorylation of YAP at S127 (Fig. 2A) and decreased phospho-YAP to total YAP ratios (Fig. 2B). Quantitative analyses of the ratio of phosphorylated to total YAP levels indicated a significant decrease between 30 and 60 min. after treatment with Jasp (Fig. 2B). By 2 h after Jasp treatment, this ratio restored to pre-treatment levels.

The phosphorylation of YAP at Ser127 retains YAP in the cytoplasm [25], [26] and treatment of cells with serum or growth factors (e.g. EGF) lead to reduced phosphorylation and nuclear localization of YAP [20], [27]. Similarly, Jasp treatment increased nuclear localization of YAP in HeLa cells (Fig. 2C). Moreover, the number of cells with nuclear YAP localization was higher after Jasp treatment as compared to untreated control group (Fig. 2D).

### Actin Polymerization Increases Expression of CCN Growth Factors

**Figure 3:**
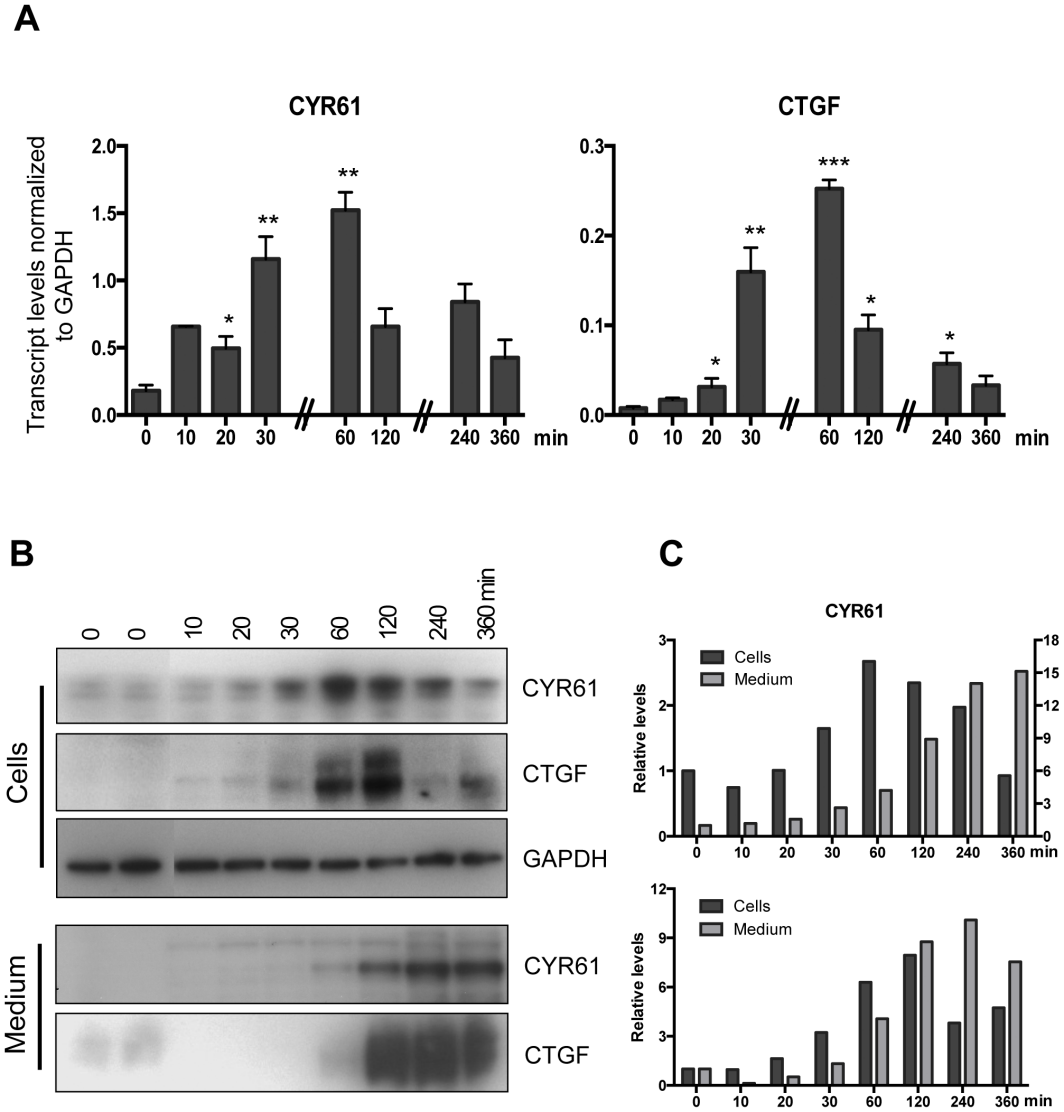
Treatment with jasplakinolide increases expression of CCN growth factors in HeLa cells. A. Transcript levels for CCN growth factors increased after jasplakinolide treatment. HeLa cells were treated with 0.5 µM jasplakinolide for 10 min. and then cultured for indicated times periods. Representative data from two independent experiments is shown. Error bars represents mean ± SEM, n = 3. *P<0.05, **P<0.001 and ***P<0.0005. B. CYR61 and CTGF protein levels after jasplakinolide treatment. HeLa cells were treated with 0.5 µM jasplakinolide for 10 min. and then cultured for indicated time periods. Protein levels for CYP61 and CTGF were measured from both medium and cells as described in Materials and Method. C. Quantitative estimation of CYR61 and CTGF antigens from both cells and medium fractions. For CYR61, the values of medium fractions are plotted on right axis.

The CCN family of secreted proteins consists of six members designated as CCN1 to CCN6. CCN family of secreted proteins are involved in various biological functions including cell proliferation, migration, survival, differentiation, and angiogenesis [28], [29]. The nuclear localized YAP has been shown to bind TEAD transcription factors and increase the transcription of CCN and other growth factors [30]. The CCN family growth factors are well-characterized targets of YAP and found to be highly expressed in cells following either overexpression of YAP [30] or by treatment with LPA to increase YAP levels [20]. As shown in Fig. 3, nuclear localization of YAP after Jasp treatment was associated with increases in transcript levels for the CCN family growth factors CCN1/CYR61 and CCN2/CTGF. The mRNA levels for these factors were increased in a time-dependent manner after Jasp treatment followed by a decline (Fig. 3A). Similar to mRNA levels, cellular content for CYR61 and CTGF proteins also increased after Jasp treatment (Fig. 3B) with a peak at 2 h after Jasp treatment followed by a decline. Subsequently, CYR61 and CTGF proteins were increased in the culture media accompanied by decreases in cellular content (Fig. 3B). Quantification of immuno-blotting results (Fig. 3C) indicated decreases in cellular content of CYR61 and CTGF, after 2 h of Jasp treatment.

### YAP Knockdown Inhibits Increases in CCN Growth Factor Expression following Jasplakinolide Treatment

**Figure 4:**
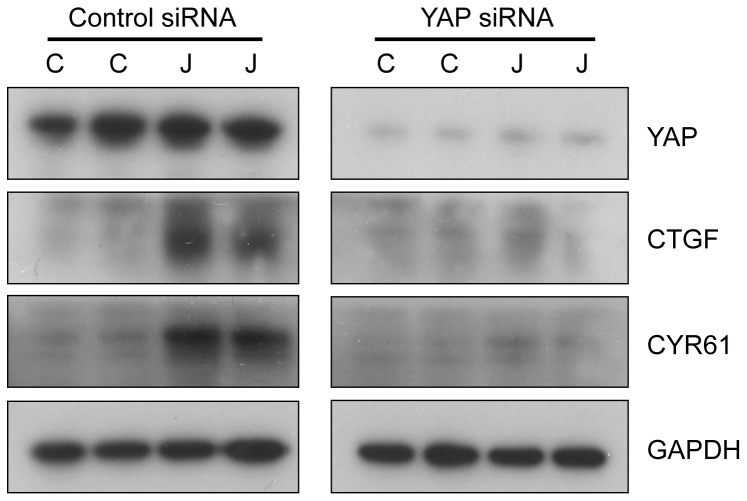
Knockdown of YAP suppresses CCN growth factor expression after actin polymerization. CYR61 and CTGF protein levels after YAP knockdown. HeLa cells were transfected with control and YAP siRNAs for 48-PAGE analyses. Immunoblotting was performed using specific antibodies.

To further determine if increases of CCN family growth factors after Jasp treatment is mediated through YAP, the levels of YAP were suppressed using siRNA (Fig. 4). The suppression of YAP inhibited increases in both CCN growth factors, CYR61 and CTGF; the protein levels of CYR61 and CTGF were not elevated after Jasp treatment (Fig. 4). The inhibition of CCN growth factors expression following by YAP suppression confirms the essential role of YAP in mediating Jasp-induced increases in CCN growth factors and demonstrate the importance of Hippo signaling in Jasp actions.

## Discussion

Although genome-wide screening studies in *Drosophila* cells demonstrated the important role of actin polymerization in Hippo signaling disruption [14], [15], most studies in mammalian cells were based on indirect measurement of F-actin levels. NIH 3T3 cells cultured on larger microdomains, to prevent cell-cell contact and adhesion showed higher F-actin staining and nuclear localized YAP [17]. Similarly, extracellular matrix (ECM) stiffness, cell detachment, and shape lead to changes in levels of F-actin, which further regulate the activity and localization of YAP in MCF10A cells [16], [18].

Under high cell density culture conditions, the levels of F-actin in the cells are low and the Hippo signaling pathway is active. In the current study using actin stabilizing drug jasplakinolide, the monomeric G-actins in the cell were converted to F-actin (Fig. 1). The bundling of actin filaments lead to the activation of YAP by reducing YAP phosphorylation (Fig. 2). The activated YAP is localized to the nucleus and induced the expression of CCN growth factors CYR61 and CTGF (Fig. 3). The phosphorylation of YAP was restored back after 1 h of Jasp treatment (Fig. 2B) and the levels of growth factors started to decrease after 1 h of Jasp treatment (Fig. 3A). Further, suppression of YAP activity inhibited the transient increases of growth factor expression after Jasp treatment (Fig. 4). In *Drosophila* studies, the effect of F-actin on Yorkie/Yki (orthologous to mammalian YAP) was shown to be mediated through Wts/LATS but not hpo/MST [15]. In addition, cell detachment and G-protein-coupled receptor signaling lead to changes in the kinase activity and phosphorylation status of LATS1, but not MST1/2 [18], [20], [31]. Moreover, LATS1-independent regulation of YAP phosphorylation by actin cytoskeleton was also reported [16], [19]. The results from our study show changes in actin polymerization can activate YAP and induce the downstream growth factors CYR61 and CTGF. The mechanism by which Hippo pathway senses the changes in the actin dynamics and the direct role of G-actin on inhibiting YAP dephosphorylation is not clear. SRF and its downstream genes are regulated by F-actin and not by G-actin. The effect of Jasp on Hippo signaling does not involve SRF, the cells pre-treated with inhibitor of SRE pathway (CCG-1423) does not block the Jasp effect on Hippo pathway (data not shown). Moreover, we do not know if Jasp could exert effects on YAP other than phosphorylation.

Jasplakinolide treatment rapidly converted the monomeric G-actin to F-actin; the levels of F-actin were maintained for long period of time (Fig. 1A). For cells to maintain their shape and stay healthy there has to be a proper equilibrium between G-actins and F-actins. Similarly C3, an inhibitor of RhoA was shown to prevent leptin induced actin polymerization and hypertrophic effects in cardiomyocytes [32]. Accordingly, increased levels of F-actin in yeast resulted in higher levels of reactive oxygen species (ROS) that caused cell death [33] and treatment of human airway epithelial cells with jasplakinolide also lead to increase in apoptosis [34]. Similarly, even though we observed increase of CCN growth factors after jasplakinolide treatment we did not observe any changes in cell proliferation (data not shown), which might be due to the prolonged retention of F-actin levels in cells (Fig. 1A, lower panel). Further, increase of F-actin levels were observed near the site of tissue injury [35], [36]. Increased levels of F-actin at site of injury due to loss of cell-cell contact might promote surrounding cells to proliferate and migrate by activating YAP, as part of the wound healing process. Results from the current study demonstrate the regulation of Hippo signaling by actin cytoskeleton, where the F-actin can activate YAP, the effector candidate of Hippo signaling and increase the expression of downstream growth factors.

## Materials and Methods

### Reagents

Antibodies to pYAP (Ser127) (#4911), YAP (#4912) and GAPDH (#2118) were obtained from Cell Signaling Technology (Beverly, MA). Jasplakinolide, CYR61 (SC-13100) and CTGF (SC-14939) were from Santa Cruz Biotechnology (Santa Cruz, CA). G-actin/F-actin in vivo assay kit was from Cytoskeleton Inc. (Denver, CO). YAP siRNA was from Qiagen (Valencia, CA).

### Cell Culture and Transfection

HeLa cells were cultured in Dulbeccós Modified Eagle Medium (DMEM, Life Technologies, Inc.) supplemented with 10% fetal bovine serum, penicillin and streptomycin. The cells were serum starved overnight and treated with jasplakinolide for 10 min. After treatment the cells were washed with medium twice and further cultured for different time periods, except for 5 and 10 min. time point during which the treatment was continuous. siRNA was transfected to cells using HiPerFect transfection reagent (Qiagen). The cells were transfected with siRNA in the complete medium and cultured for 48 h.

### Immunoblotting

HeLa cells were cultured to confluency and were serum-starved overnight before treatment with jasplakinolide. The cells were treated with jasplakinolide for 10 min. and washed twice with serum-free medium before further culture. Before immunoblotting, cells were lysed in M-PER lysis buffer supplemented with protease inhibitors. The cell lysates were run on SDS-PAGE and transferred to PVDF membranes. To measure secreted proteins in the medium, culture media from the wells were collected and concentrated using centrifugal filter units (Millipore). The reducing sample buffer was added to the final concentrated media and run on SDS-PAGE. Densitometry analysis was done using ImageJ (National Institute of Health, Bethesda, MD).

### Real-time PCR

Total RNA was extracted from cells using RNeasy kit (Qiagen). The first strand synthesis was done using Sensiscript RT kit (Qiagen) and quantitative PCR was performed in triplicates using iTaq SYBR Green Supermix kit (Bio-Rad). Transcript levels were normalized based on GAPDH levels. The primers used are: hCYR61: AGCCTCGCATCCTATACAACC and TTCTTTCACAAGGCGGCACTC, hCTGF: GCGTGTGCACCGCCAAAGAT and CAGGGCTGGGCAGACGAACG, hYAP: TCAGACAACAACATGGCAGGA and TTCATGGCTGAAGCCGAGTT, hGAPDH: GGTGAAGGTCGGAGTCAAC and CCATGGGTGGAATCATATTG.

### Immunofluorescence

Cells were grown on cover slips. After jasplakinolide treatment the cells were fixed in 4% paraformaldehyde and permeabilized with 0.1% Triton x-100. The primary and secondary antibodies were diluted in the blocking buffer (1% BSA/PBS). Cells showing nuclear YAP staining was quantified by counting the cells from five different fields. The mean of each treatment group from two independent experiments were plotted. Hoechst 33342 (Invitrogen) was used for staining the nucleus and the coverslips were mounted on microscope slides using Vectashield mounting medium (Vector shield).

### G-actin/F-actin Assay

The amount of G-actin and F-actin in HeLa cells was quantified using G-actin/F-actin in vivo assay kit according to the manufacturer instructions. Briefly, the cells were lysed in pre-warmed lysis/F-actin stabilizing buffer supplemented with protease inhibitor and ATP. The cell lysate was centrifuged for 5 min. at 350×g to remove the cell debris. Cell lysate (100 ul) was ultracentrifuged at 100,000×g for 1 h at 37°C to pellet the F-actin with G-actin remaining in the supernatant. F-actin in the pellet was resuspended in 100 ul of F-actin destabilizing buffer on ice for 1 h with frequent pipetting. Equal volumes of G-actin and F-actin fractions were mixed with 5× SDS sample buffer and run on SDS-PAGE. Western blot was done using anti-actin antibody provided in the kit. Densitometry analysis was done using ImageJ.

### Statistical Analysis

All experiments were repeated at least three times. For comparisons the differences between groups were calculated with Student’s t-test, and a difference was considered significant if *P*<0.05.
