# Correction: Actin Cytoskeleton Regulates Hippo Signaling

**DOI:** 10.1371/annotation/ea0f1c59-8ed0-4870-9dab-7afd2ca63328

**Published:** 2013-09-24

**Authors:** Pradeep Reddy, Masashi Deguchi, Yuan Cheng, Aaron J. W. Hsueh

There were errors in the affiliations presented in the article. The correct author list and affiliations are available below.

Pradeep Reddy^1,2^, Masashi Deguchi^1^, Yuan Cheng^1^, Aaron J. W. Hsueh^1*^

1 Program of Reproductive and Stem Cell Biology, Department of Obstetrics/Gynecology, Stanford University School of Medicine, Stanford, California, United States of America,

2 Current address: Gene Expression Laboratory, Salk Institute for Biological Studies, La Jolla, California, United States of America 

